# Comprehensive analysis of pivotal biomarkers, immune cell infiltration and therapeutic drugs for steroid-induced osteonecrosis of the femoral head

**DOI:** 10.1080/21655979.2021.1972081

**Published:** 2021-09-07

**Authors:** Bo Wang, Song Gong, Wenkai Shao, Lizhi Han, Zilin Li, Zhichao Zhang, Yang Zheng, Fang Ouyang, Yan Ma, Weihua Xu, Yong Feng

**Affiliations:** a Department of Rehabilitation, Wuhan No. 1 Hospital, Tongji Medical College, Huazhong University of Science and Technology, Wuhan China; b Department of Orthopedics, Union Hospital, Tongji Medical College, Huazhong University of Science and Technology, Wuhan China

**Keywords:** SONFH, immune cell infiltration, biomarker, therapeutics

## Abstract

Steroid-induced osteonecrosis of the femoral head (SONFH) is a progressive disease that leads to an increased disability rate. This study aimed to ascertain biomarkers, infiltrating immune cells, and therapeutic drugs for SONFH. The gene expression profile of the GSE123568 dataset was downloaded from the Gene Expression Omnibus (GEO) database. The differentially expressed genes (DEGs) were identified using the NetworkAnalyst platform. Functional enrichment, protein-protein interaction network (PPI), and module analyses were performed using Metascape tools. An immune cell abundance identifier was used to explore immune cell infiltration. Furthermore, hub genes were identified based on maximal clique centrality (MCC) evaluation using cytoHubba application and confirmed by qRT-PCR using clinical samples. Finally, the L1000 platform was used to determine potential drugs for SONFH treatment. The SONFH mouse model was used to determine the therapeutic effects of aspirin. In total, 429 DEGs were identified in SONFH samples. Functional enrichment analysis showed that these DEGs were enriched in myeloid leukocyte activation and osteoclast differentiation processes. A set of nine immune cell types was confirmed to be markedly different between the SONFH and control samples. All 10 hub genes were significantly highly expressed in the serum of SONFH patients, as shown by qRT-PCR. Finally, the therapeutic effect of aspirin on SONFH was examined in animal experiments. Taken together, our data revealed the hub genes and infiltrating immune cells in SONFH, and we also screened potential drugs for use in SONFH treatment.

## Introduction

Glucocorticoids are widely used to treat connective tissue and inflammatory disorders, such as systemic lupus erythematosus, Sjogren’s syndrome, rheumatoid arthritis, and severe acute respiratory syndrome [1,2]. However, one of the most common complications following administration of steroids is steroid-induced osteonecrosis of the femoral head (SONFH) [3]. Recent studies have shown that glucocorticoids are the primary risk factor for nontraumatic ONFH in both China and Japan [4,5]. SONFH is a progressive disease, and when untreated, it can spark the devastation and dysfunction of hip joints and eventually impair quality of life. As a consequence of this disease, more than half of the patients require artificial joint replacement. Regretfully, the pathogenesis of this disease is unclear. Therefore, exploring the molecular etiological factors and discovering novel biomarkers for early diagnosis and individualized therapy are urgently needed.

Apoptosis, necrosis, pyrolysis of osteoblasts and osteocytes, intravascular fat embolism, elevated intraosseous pressure, circulatory impairment, coagulation disorders, and genetic polymorphisms have been confirmed to play crucial roles in the pathogenesis of SONFH [6–10]. Several studies have focused on the role of inflammatory reaction in the development of ONFH [11,12]. It has been found that abnormal immune cell infiltration, inflammatory pathway activation, and cytokine release might be associated with the pathophysiology of ONFH [13,14]. However, few studies have investigated immune cell infiltration of SONFH. Thus, exploring the distribution of immune cells infiltrating the femoral head in SONFH may contribute to the clarification of the pathogenesis of SONFH.

Bioinformatics is an emerging interdisciplinary field involving molecular biology and information science [15]. A large number of researchers have performed chip sequencing or high-throughput sequencing experiments and uploaded the results to openly accessed repositories, such as the ArrayExpress and Gene Expression Omnibus (GEO) database [16,17]. In our study, we downloaded the primitive microarray dataset GSE123568 from the GEO database and analyzed it using bioinformatics tools. Moreover, immune cell infiltration in SONFH samples was evaluated using the ImmuCellAI method, which is applied to evaluate the abundance of 24 immune cell types. Moreover, the expression levels of key genes were examined using qRT-PCR. Additionally, drugs with potential therapeutic effects on SONFH were predicted based on the differentially expressed genes (DEGs) identified. Ultimately, the effects of aspirin were validated in SONFH treatment using a glucocorticoid-mediated mouse model of ONFH.

Thus, the aim of our research was to identify crucial biomarkers of DEGs and immune infiltration in SONFH, and explore drugs with potential therapeutic effects on SONFH. Eventually, novel diagnostic and therapeutic measures should be established for SONFH.

## Materials and methods

### Microarray data and identification of DEGs

The microarray data of the GSE123568 dataset were downloaded from the GEO database (GPL15207 platform, Affymetrix Human Gene Expression Array). GES123568 comprises 40 samples of 30 peripheral serum samples obtained from SONFH patients and 10 peripheral serum samples from healthy patients.

NetworkAnalyst (https://www.networkanalyst.ca/), an online tool [18], was used to explore the DEGs by comparing the expression values between SONFH patient samples and normal control samples. In the present study, an adjusted *p* value less than 0.01 and log FC value greater than 1.0, were selected as the cutoff criteria to identify DEGs. Additionally, we generated a volcano map based on adjusted *p* and log FC values. Moreover, we applied a hierarchical clustering analysis method to evaluate the DEGs (50 genes are shown) between SONFH samples and control samples, and the results were visualized using NetworkAnalyst.

### Function enrichment analysis and PPI network construction

Metascape (http://metascape.org) is a free-of-charge, data-rich, and well-maintained online tool for gene annotation and analysis [19]. It can provide gene enrichment information according to biological categories, including biological process (BP), cellular component (CC), molecular function (MF), and pathway enrichment annotation to deliver exhaustive and elaborate information on each gene. Therefore, in this study, Gene Ontology (GO) terms, consisting of BP, CC, MF, and Kyoto Encyclopedia of Genes and Genomes (KEGG) pathways, were used to evaluate enriched genes in Metascape. Only terms with *p* < 0.01, a minimum count of 3, and an enrichment factor > 1.5 were considered significant. Protein – protein interaction (PPI) enrichment analysis was performed using Metascape. Furthermore, the molecular complex detection (MCODE) algorithm was used to investigate highly related network units.

### Immune cell infiltration as determined via ImmuCellAI analysis

The ImmuCellAI (http://bioinfo.life.hust.edu.cn/web/ImmuCellAI/) algorithm [20] was used to assess the abundance of 24 immune cell types in SONFH and normal samples. These immune cells include 18 T cell subsets and six other critical immune cells, including B cells, macrophages, monocytes, neutrophils, dendritic cells (DCs), and natural killer (NK) cells [17]. We first uploaded the gene expression files (GSE123568) obtained previously. Then, immune cell abundance in the groups was set up for analysis selection. The abundance of each immune cell type in the two groups was calculated using this method.

### Hub genes identification and validation in vitro

Hub genes (10 most expressed genes) were obtained based on maximal clique centrality (MCC) evaluation by cytoHubba, a plugin in Cytoscape software [21]. Peripheral serum samples from six SONFH patients and six healthy control adults were harvested for qRT-PCR validation. The study protocol was approved by the Research Ethics Commission of Wuhan Union Hospital, and informed consent was obtained from all participants.

### Quantitative real-time-PCR (qRT-PCR)

The mRNA transcripts were quantified by qRT-PCR using AceQ® Universal SYBR qPCR Master Mix (Vazyme Biotech, Q511-02) and a quantitative real-time PCR system (StepOnePlus, ABI, USA). The amplification conditions were set as follows: 94°C for 4 min, followed by 35 cycles at 94°C for 20 s, 60°C for 30 s, and 72°C for 30 s. Then, a melting curve was established to obtain the experimental data. GAPDH was used as the reference gene, and all experiments were conducted in triplicates. Relative target gene expression levels were calculated using the 2^−ΔΔCt^ method. The primers used in this study are listed in Supplementary Table S1.

### Prediction of potential therapeutic drugs

A connectivity map (https://clue.io/query) was used to identify potential therapeutic drugs for SONFH based on uploaded DEG data. This connectivity map, an online tool, is used to compare queried signatures with a gene expression profile database of several cell lines treated with more than 2000 compounds, most of which have been approved by the U.S. Food and Drug Administration [22]. Compounds with therapeutic potential usually affect biological processes opposite of the disease. Thus, these compounds may have certain effects.

### Animals and experimental procedures

The research protocol was approved by the Committee on Ethics of Animal Experiments of the Wuhan Union Hospital. Male BALB/c mice weighing 18.2–26.1 g (8 weeks) were purchased from the Laboratory Animal Center of Huazhong University of Science and Technology (Wuhan, China). They were then randomly divided into three groups: the control group, glucocorticoid (GC) group, and GC+ aspirin group, with 5 mice in each group. The SONFH model was induced by treating the mice with 2 mg/ml dexamethasone (Dex, National Medicine Standard H41020055, Zhengzhou Cheuk-Fung Pharmaceutical Company, China) administered in drinking water for 8 weeks. For the GC+ aspirin treatment group, mice were treated with aspirin (10 mg/kg) daily via gavage. The diagnosis of osteonecrosis was established based on the presence of empty lacunae or osteocytes with pyknotic nuclei in the bone trabeculae, accompanied by surrounding bone marrow necrosis [23]. At the end of the treatment, mice were sacrificed and samples of the femoral head were obtained for RNA extraction and histological examination. For histological examination, femoral head samples were decalcificated, paraffin embedded, sectioned and stained with hematoxylin and eosin or TRAP staining kit using standard procedures [24,25]. For RNA extraction, femoral head samples were collected, immediately snaped frozen in liquid nitrogen, and RNA was extracted as described above.

### Statistical analysis

All results are presented as the mean ± standard deviation (SD) from at least three independent experiments. Statistical analysis was carried out using SPSS software (version 23.0). Statistical significance was determined using a two-tailed Student’s t-test when two groups were compared, and one-way ANOVA was followed by Bonferroni’s post hoc test when comparing more than two groups. GraphPad Prism 7 software was used for drawing the diagrams. A *p* value lower than 0.05, was considered to represent a statistically significant difference.

## Results

### Identification of DEGs

**Figure 1.**
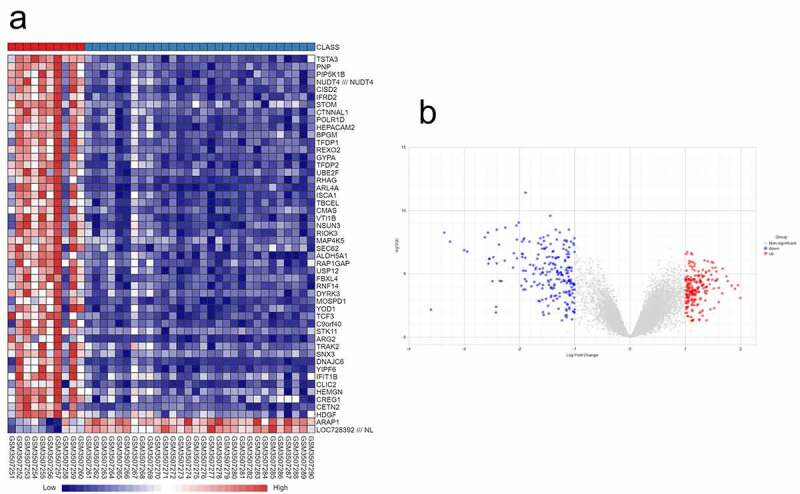
Identification of DEGs in SONFH. (a) Hierarchical clustering heat map of SONFH samples and control samples based on identified DEGs (50 genes shown). (b) Volcano plot of the DEGs between SONFH samples and control samples

Following the search keywords ‘osteonecrosis of the femoral head,’ ‘glucocorticoid’ and ‘expression profiles by array’ we obtained the GSE123568 dataset from the GEO database. A total of 429 DEGs (Supplementary Table S2), including 214 upregulated genes and 215 downregulated genes, were identified in the SONFH samples compared with the normal controls using NetworkAnalyst. A volcano diagram and heat map of the DEGs are shown in Figure 1.

### Functional enrichment analysis and PPI network construction

**Figure 2.**
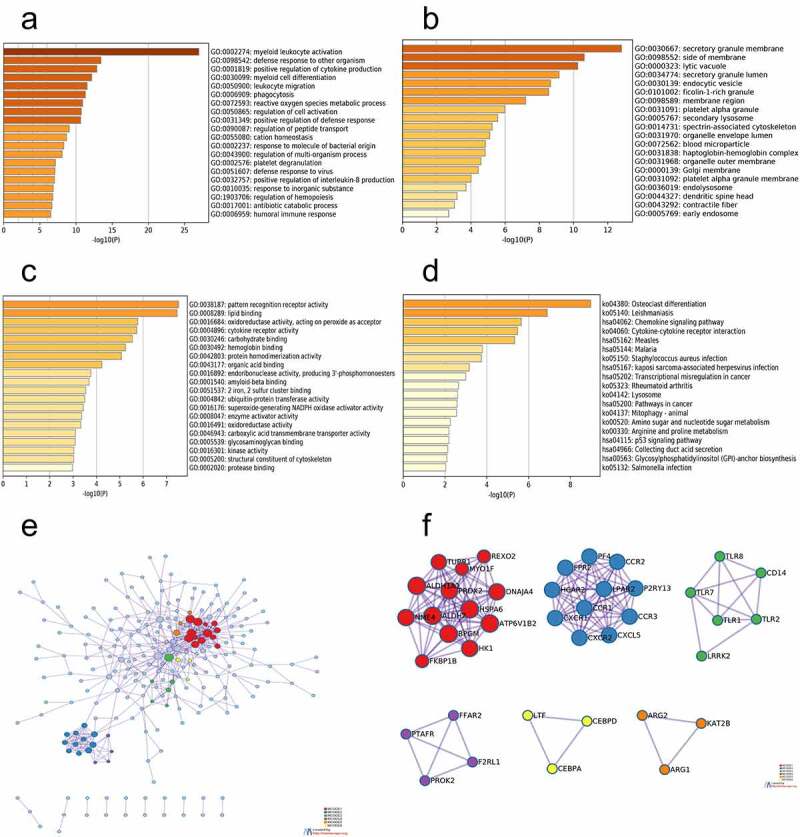
Function enrichment analysis and PPI network construction. (a) GO functional enrichment analysis of BP. (b) GO functional enrichment analysis of CC. (c) GO functional enrichment analysis of MF. (d) KEGG pathway analyses results of the DEGs. (e) The PPI network of the DEGs. (f) The six most significant modules were obtained from the PPI network

The functions of the DEGs were assessed by conducting GO and KEGG analyses in Metascape. GO functional enrichment analysis in the BP category revealed that the DEGs were mainly involved in myeloid leukocyte activation, defense response to other organisms, positive regulation of cytokine production, and myeloid cell differentiation (Figure 2a). In the CC category, the DEGs were mainly enriched in the secretory granule membrane, side of membrane, lytic vacuole, and secretory granule lumen (Figure 2b). In addition, in the MF category, the DEGs were predominantly enriched in pattern recognition receptor activity, lipid binding, oxidoreductase activity, and cytokine receptor activity (Figure 2c). KEGG pathway analysis also demonstrated that the DEGs were notably enriched in osteoclast differentiation, leishmaniasis, chemokine signaling pathway, and cytokine-cytokine receptor interaction (Figure 2d). In addition, to better explain the correlation among each DEG, a Metascape protein–protein interaction (PPI) enrichment analysis was performed. The PPI network and MCODE components in the gene list are illustrated in Figure 2e, f. The six most significant MCODE components were extracted from PPIs. After pathway and process enrichment analysis, which was independently performed for each MCODE component, the results revealed that the biological functions were chiefly associated with protein refolding, GPCR ligand binding, MyD88-dependent Toll-like receptor signaling pathway, G alpha (q) signaling events, negative regulation of cell proliferation, and regulation of osteoblast differentiation (Table 1).

**Table 1.** The six most significant MCODE components were extracted from the PPI

MCODE_ALL	R-HSA-373076 Class A/1 (Rhodopsin-like receptors); R-HSA-500792 GPCR ligand binding; GO:0002274 myeloid leukocyte activation
MCODE_1	GO:0042026 protein refolding; GO:0019320 hexose catabolic process; hsa00010 Glycolysis/Gluconeogenesis
MCODE_2	R-HSA-373076 Class A/1 (Rhodopsin-like receptors); R-HSA-418594 G alpha (i) signaling events; R-HSA-500792 GPCR ligand binding
MCODE_3	GO:0002755 MyD88-dependent toll-like receptor signaling pathway; GO:0032757 positive regulation of interleukin-8 production; GO:0032677 regulation of interleukin-8 production
MCODE_4	R-HSA-416476 G alpha (q) signaling events; R-HSA-373076 Class A/1 (Rhodopsin-like receptors); R-HSA-500792 GPCR ligand binding
MCODE_5	GO:0008285 negative regulation of cell proliferation
MCODE_6	GO:0045669 positive regulation of osteoblast differentiation; GO:0045778 positive regulation of ossification; GO:0045667 regulation of osteoblast differentiation

### Immune cell infiltration analysis

**Figure 3.**
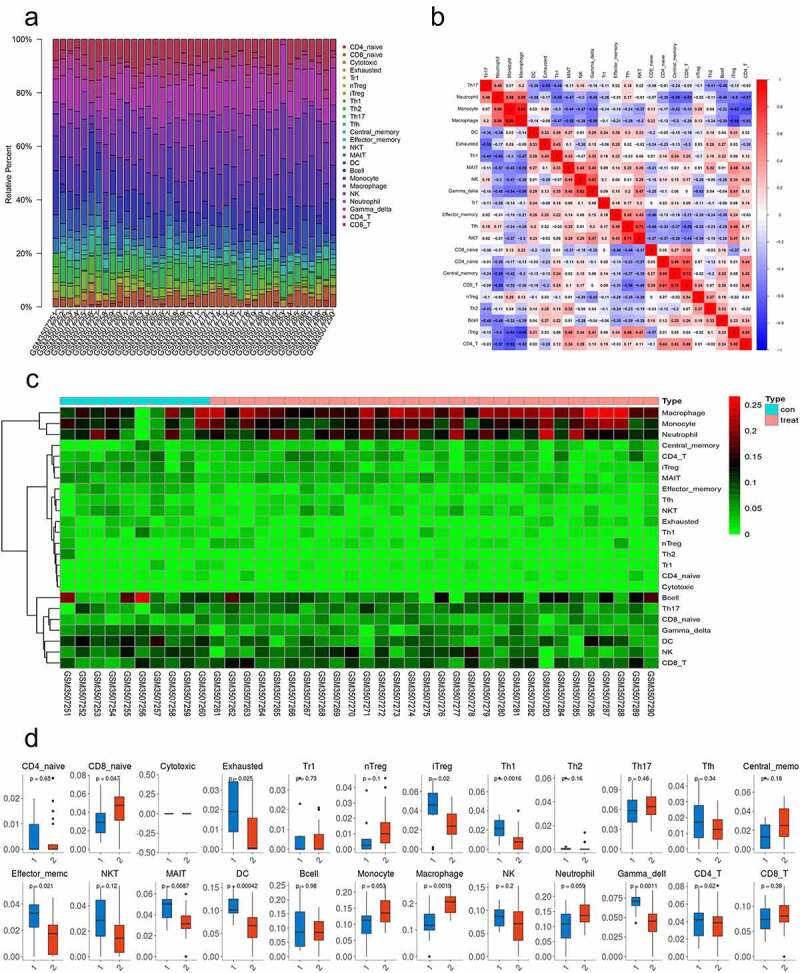
The differences in immune cell infiltration between SONFH and normal controls. (a) The relative percentage of 24 subpopulations of immune cells in 40 samples from the GSE123568 dataset. (b) Correlation analyses of the 24 subpopulations of immune cells estimated in 40 samples from GSE123568. (c) Heat map of the 24 subpopulations of immune cells estimated in 40 samples from GSE123568. (d) The differences in immune cell infiltration between SONFH patients and normal controls (*p values < 0.05 were considered statistically significant)

Due to scientific and technological limitations, the pattern of immune cell infiltration in SONFH has not been completely clarified, particularly in subgroups consisting of few cells. By utilizing the ImmuCellAI algorithm, we first investigated the differences in immune cell infiltration among 24 subpopulations of immune cells in these two groups (Figure 3a, b). Compared with the control group, the SONFH group displayed an elevated percentage of naive CD8 + T cells and macrophages, whereas the proportions of exhausted T cells, induced regulatory T cells (iTregs), T helper 1 cells (Th1 cells), effector memory T cells, mucosal-associated invariant T cells (MAIT cells), DCs, and gamma delta T cells were lower (Figure 3c, *p* < 0.05). Furthermore, we investigated the correlation between these types of immune cells. Macrophages were highly correlated with monocytes (Pearson correlation = 0.86), indicating that the functions of macrophages and monocytes may be synergistic (Figure 3d).

### Hub gene identification and validation in vitro

**Figure 4.**
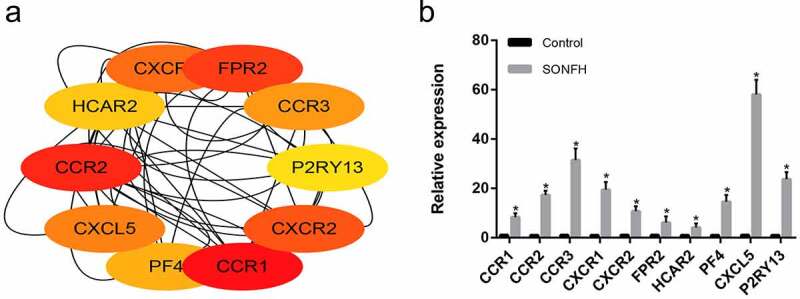
The 10 most highly expressed hub genes in the PPI network as confirmed with RT-PCR. (a) The 10 most highly expressed hub genes in the PPI network as determined by cytoHubba. (b) RT-PCR validation of the hub genes in SONFH and normal controls. All experiments were performed in triplicate, and the results are presented as the means ± SD. (*p < 0.05)

Based on the MCC centrality evaluation by cytoHubba, the 10 most highly expressed hub genes *CCR1, CCR2, CCR3, FPR2, CXCR2, CXCR1, CXCL5*, PF4, *HCAR2*, and *P2RY13*, were identified (Figure 4a). We then detected the expression of these 10 hub genes in six SONFH and six control samples via qRT-PCR. The results illustrated that the comparative expression levels of all 10 hub genes were in line with the microarray hybridization results (Figure 4b).

### Prediction of potential therapeutic drugs for SONFH

To identify potentially therapeutically valuable compounds that may be the most suitable for targeting DEGs, we uploaded DEGs to the connectivity map L1000 platform, a tool used to compile gene expression profiles associated with a variety of therapeutic compounds. The 10 compounds most closely associated with DEGs are listed in Table 2 with their gene targets, including phylloquinone, cholic acid, MRS-1220, bucladesine, isotretinoin, doxercalciferol, betahistine, aspirin, maraviroc, and SR-27897. Among the target genes, we found that *PTGS2*, a gene target of aspirin, was significantly upregulated in the GSE123568 dataset, indicating a valuable role for aspirin in SONFH treatment.

**Table 2.** The top 10 compounds with activity against SONFH as predicted via connectivity map

Score	Name	Description	Target
−99.68	Phylloquinone	Vitamin K	BGLAP, GGCX
−99.58	cholic-acid	Bile acid	ADH1C, CES1, COX4I1, COX5A, COX5B, COX6A2, COX6B1, COX6C, COX7A1, COX7B, COX7C, COX8A, ESRRG, FABP6, FECH, GPBAR1, MT-CO1, MT-CO2, MT-CO3, PLA2G1B
−99.58	MRS-1220	Adenosine receptor antagonist	ADORA2B, ADORA3
−99.54	Bucladesine	Adenosine receptor agonist	PRKACA
−99.51	Isotretinoin	Retinoid receptor agonist	CYP2B6, CYP2C19, CYP2C8, CYP3A5, CYP3A7, NR2C2, PPARD, RARA, RARB, RARG, RORB
−99.22	Doxercalciferol	Vitamin D receptor agonist	VDR
−98.8	Betahistine	Histamine receptor agonist	HRH1, HRH3
−98.8	Aspirin	Cyclooxygenase inhibitor	AKR1C1, ASIC3, EDNRA, HSPA5, IKBKB, NFKB1, NFKB2, NFKBIA, PRKAA1, PRKAA2, PRKAB1, PRKAB2, PRKAG1, PRKAG2, PRKAG3, PTGS1, PTGS2, RPS6KA3, TP53
−98.73	Maraviroc	CC chemokine receptor antagonist	CCR5, CYP3A5
−98.38	SR-27897	CCK receptor antagonist	CCKAR

**Figure 5.**
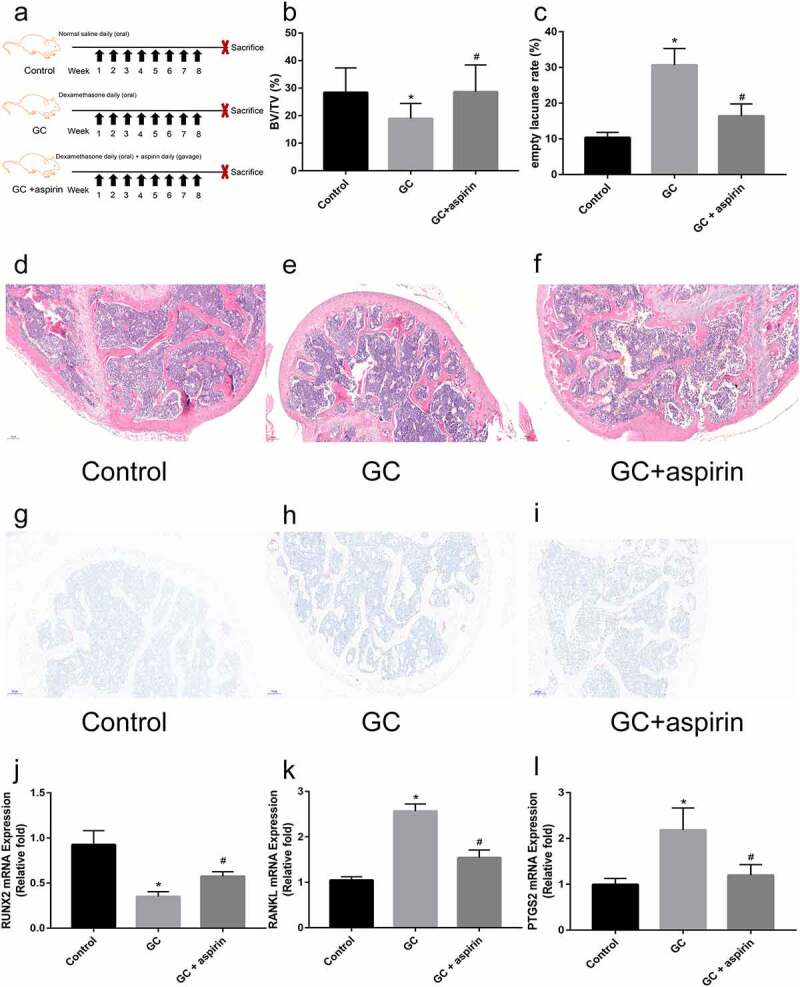
Identification of the therapeutic effect of aspirin in GC-induced ONFH. (a) Schematic diagram of the treatment of different groups of mice. H&E staining of representative femoral heads in each group. (b) Bone volume/total volume ration (BV/TV) in each group. (c) Empty lacunae rate in each group. (d-f) H&E staining of representative femoral heads in each group (scale bar = 100 μm). (g-i) TRAP staining of representative femoral heads in each group (scale bar = 100 μm). (j-l) Relative *Runx2, RANKL* and *PTGS2* mRNA expression in femoral heads in each group. All experiments were performed in triplicate, and the results are presented as the means ± SD. (*p < 0.05 versus the control; #*p* < 0.05 versus the GC)

A mouse model of glucocorticoid (GC)-induced ONFH was generated to further explore the role of aspirin in the treatment of SONFH (Figure 5a). Compared with mice in the GC treatment group, GC+ aspirin treatment ameliorated GC-induced ONFH in mice by increasing bone volume/total volume (BV/TV) and reducing the empty lacunae rate (Figure 5b-f). However, aspirin treatment did not inhibit osteoclastogenesis in GC-induced ONFH in mice. As shown in Figure 5g-I, compared with that in the GC treatment group, the number of osteoclasts in the femoral head tissue in the GC+ aspirin treatment group was not significantly reduced by TRAP staining. Furthermore, relative Runx2 mRNA expression in the femoral head tissue was significantly increased by aspirin treatment (Figure 5j). Relative RANKL mRNA expression in the femoral head tissue was significantly decreased by aspirin treatment, compared with the GC treatment group (Figure 5k). Since PTGS2 was predicted to be the target gene of aspirin, PTGS2 mRNA expression was detected by qRT-PCR in different groups of mice. The results revealed that aspirin treatment reversed GC treatment-induced increase in PTGS2 expression (Figure 5l). Taken together, these results indicate that aspirin may be a drug for the treatment of SONFH.

## Discussion

The medical history of glucocorticoid use and related clinical manifestations, such as pain in the hip joint, and imaging examinations, including those on plain film, bone scintillation, and magnetic resonance imaging, are often used for the diagnosis of SONFH [26]. However, symptoms may not appear in the early stages of the disease, but rather in the middle and late stages. Because the disease cannot be diagnosed early, the hip preservation treatment is delayed. Although magnetic resonance imaging is often used as a method for the early diagnosis of this disease, it is not suitable for screening all patients receiving GC therapy because of the high cost and time commitment required for obtaining scans. Thus, identifying key circulating markers is essential for the early diagnosis of SONFH. In recent years, some pivotal biomarkers for SONFH have been explored, such as type I collagen cross-linked C-telopeptide and amino terminal telopeptide of procollagen type I [27]. However, both two genes were found to be associated with the development of osteoporosis, which limited their diagnostic value [28]. Therefore, it is necessary to identify promising biomarkers for SONFH to improve its diagnosis and treatment. In our study, we attempted to identify potentially crucial genes related to SONFH. A bioinformatics assessment of GO and KEGG enrichment analyses was performed, and pivotal hub genes were identified in our study. Moreover, we adopted the ImmuCellAI tool to identify immune cell infiltration in SONFH. The results showed a remarkable difference in immune cell infiltration between the SONFH and control samples.

In the present study, we discovered that the DEGs were mostly enriched in myeloid leukocyte activation, positive regulation of cytokine production, myeloid cell differentiation, and cytokine receptor activity. Previous studies have verified that the cytokines IL-10, IL-12, and TNF-α are related to the development of ONFH [29]. In addition, osteoprotegerin, receptor activator of nuclear factor-κB and receptor activator of nuclear factor-κB ligand (RANKL), a cytokine/cytokine receptor signal axis, have been confirmed to regulate the balance between osteogenesis and osteoclastogenesis, which has also been found to be associated with the pathophysiology of ONFH [30]. Additionally, the outcomes of KEGG pathway analysis revealed that DEGs were notably enriched in osteoclast differentiation, chemokine signaling pathways, and cytokine-cytokine receptor interactions. Moreover, six of the most significant MCODE components extracted from the PPI network were also enriched in the MyD88-dependent Toll-like receptor signaling pathway and in cell proliferation and osteoblast differentiation regulatory pathways. Two studies demonstrated that the immune response associated with the Toll-like receptor 4 signaling pathway can lead to SONFH [31,32]. In addition, abnormal osteoclast differentiation of marrow mesenchymal stem cells (MSCs) has been confirmed to be significantly associated with the development of SONFH [33–35]. Thus, we hypothesize that the immune response associated with cytokine and chemokine signaling pathways and osteoclast and osteoblast differentiation may underlie the potential pathophysiology of SONFH.

Ten crucial hub genes related to SONFH were identified from the PPI network, namely, *CCR1, CCR2, CCR3, CXCR1, FPR2, HCAR2*, PF4, *CXCR2, CXCL5*, and *P2RY13*, using the Cytoscape plugin named CytoHubba. In addition, we collected six serum samples from patients with SONFH and six control serum samples to explore the expression of 10 key pivotal genes and found similar trends with their expression levels in the GEO datasets, which confirmed the reliability of our findings. Previous studies have indicated the role of some hub genes in bone and joint diseases. For instance, *CCR2* participates in chondrocyte degradation and leads to the progression of osteoarthritis [36,37]. In addition, it has been demonstrated that overexpression of *CCR3* increases the recruitment of circulating monocytes in bone, enhances monocyte differentiation into osteoclasts, and eventually promotes the development of osteoporosis [38]. Furthermore, although CXCR1 and CXCR2 are members of a family of chemokine receptors, they seem to play opposite roles in osteoarthritis. Some researchers have pointed out that CXCR1/2 signaling can maintain cartilage homeostasis by increasing extracellular matrix production and inhibiting chondrocyte apoptosis [39,40]. As for PF4 and CXCL5, PF4 is also called CXCL4, yet their role has not been reported. CXCL5 inhibits osteoclastogenesis of MSCs [41]. However, the critical role of several key genes screened in SONFH remains unclear, such as *CCR1, FPR2, HCAR2*, and *P2RY13*. Further research is required to determine the exact roles of these hub genes in SONFH. We believe that these results can provide useful knowledge for improving the accuracy of diagnosis and treatment in patients with SONFH.

From the analysis of immune cell infiltration, we also found significant differences in the abundance of several immune cells, such as naive CD8 + T cells and macrophages. The effects of several types of immune cells in SONFH have also been explored. For example, previous studies have shown a high correlation between increased numbers of macrophages and glucocorticoid administration in patients with SONFH, which demonstrated that macrophage infiltration might be one of the causes of SONFH [42]. In addition, inflammatory infiltration of macrophages and CD4 + T cells was also observed in patients with SONFH [14]. However, the role of naive CD8 + T cell infiltration in SONFH requires further study. We also investigated the correlation between these immune cells. Macrophages were highly correlated with monocytes. Immune cell infiltration analysis showed that monocyte levels were higher in the SONFH group than in the control group, although the *p* value was slightly greater than 0.05 (p = 0.053). These results reveal that macrophages and monocytes have a synergistic effect in the development of SONFH. Many researchers have reported that macrophages and monocytes are precursors of osteoclasts [43,44]. Osteoclast differentiation-mediated bone resorption is one of the causes of femoral head collapse [45]. Hence, we speculate that high-dose glucocorticoids might activate macrophage and monocyte systems in the blood, which will infiltrate the femoral head tissues, increasing local osteoclast differentiation and leading to the destruction of the balance between bone formation and bone resorption.

In the past few decades, conservative treatment of SONFH has mainly focused on preventing collapse of the femoral head. Administration of bisphosphonate was reported to prevent collapse in early stage ONFH in a small group of patients [46]. However, the prolonged anti-collapse effects of bisphosphonates have not been elucidated. Once the femoral head progresses and collapses, surgery is needed, including hip-preserving and hip replacement surgeries. However, surgical treatment often results in greater trauma and higher costs. Therefore, it is necessary to identify novel agents and targets for the early treatment of SONFH. In this study, we screened 10 potential drugs for the treatment of SONFH. Most of them have been proven to be associated with the maintenance of normal bone metabolism and have potential therapeutic effects in SONFH. Considering the target genes of these compounds, aspirin, which targets PTGS2, attracted our attention. Because of its safety, aspirin is often used clinically as an antipyretic, analgesic, and anti-inflammatory drug. Previous studies have demonstrated its potential effects on postmenopausal osteoporosis [47]. Aspirin can inhibit osteoclast formation and improve osteogenesis by affecting a variety of biological pathways, such as inhibiting the activities of COX2, COX1, and PGE2 [48]. Furthermore, some studies have shown that aspirin may be a simple and effective treatment option to delay the progression of early ONFH in patients [49]. In this study, a mouse model of Dex-induced ONFH was developed to further explore the role of aspirin in SONFH treatment, and the results revealed that aspirin could protect against GC-induced SONFH, indicating the therapeutic effect of aspirin in SONFH. However, our results demonstrated that aspirin did not ameliorate SONFH by inhibiting osteoclastogenesis.

Although several critical hub genes, types of infiltrating immune cells, and potential therapeutic agents for SONFH were identified in our study, there are some limitations to our study. First, the sample size was relatively small, which may have led to low statistical power for examining gene expression differences between the two groups. With the establishment of the steroid-induced femoral head necrosis specimen library, we will collect more specimens for testing related indicators in the future to reduce the error caused by the small sample size. Second, although we confirmed in a mouse model that aspirin could inhibit the occurrence of SONFH, follow-up clinical trials are still needed to confirm its clinical efficacy. Finally, further studies on the molecular mechanism by which aspirin inhibits SONFH are needed.

## Conclusion

In summary, 429 DEGs were identified between the SONFH and the control groups. Analysis of the biological functions and pathways of these DEGs contributed to a better understanding of the molecular mechanism of SONFH. Ten pivotal hub genes in SONFH were identified, and the difference in infiltrating immune cells between SONFH and normal control samples was explored. In addition, considering the DEGs identified, we successfully predicted that 10 potential drugs might have therapeutic effects on SONFH. Finally, the role of aspirin in SONFH treatment was verified using a mouse model of DEX-induced ONFH. Overall, our findings might provide novel clues for investigating the pathogenesis and treatment of SONFH.

## Supplementary Material

Supplemental Material

## Data Availability

All the data in our study will be made available by the authors upon reasonable request. https://www.ncbi.nlm.nih.gov/geo/query/acc.cgi?acc=GSE123568

## Highlights

1. Ten hub genes and infiltrating immune cells were identified in SONFH.
2. Myeloid leukocyte activation may be the causes of SONFH.
3. Aspirin may be a novel treatment for SONFH.

## Abbreviations

SONFH, steroid-induced osteonecrosis of the femoral head; GEO, Gene Expression Omnibus; DEGs, differentially expressed genes; PPI, protein-protein interaction network; BP, biological process; CC, cellular component; MF, molecular function; KEGG, Kyoto Encyclopedia of Genes and Genomes; MCODE, molecular complex detection; MCC, maximal clique centrality; GC, glucocorticoid; DEX, dexamethasone; MSCs, marrow mesenchymal stem cells; RANKL, receptor activator of nuclear factor-κB ligand.

## References

[1] Hao C, Yang S, Xu W, et al. MiR-708 promotes steroid-induced osteonecrosis of femoral head, suppresses osteogenic differentiation by targeting SMAD3. Sci Rep. 2016;6(1):22599. 26932538 10.1038/srep22599 PMC4773864

[2] Huang Z, Cheng C, Cao B, et al. Icariin Protects against Glucocorticoid-Induced Osteonecrosis of the Femoral Head in Rats. Cell Physiol Biochem. 2018;47(2):694-706. 10.1159/000490023 29794448

[3] Weinstein RS, Hogan EA, Borrelli MJ, et al. The Pathophysiological Sequence of Glucocorticoid-Induced Osteonecrosis of the Femoral Head in Male Mice. Endocrinology. 2017;158(11):3817–3831. 28938402 10.1210/en.2017-00662 PMC5695837

[4] Zhao DW, Yu M, Hu K, et al. Prevalence of Nontraumatic Osteonecrosis of the Femoral Head and its Associated Risk Factors in the Chinese Population: results from a Nationally Representative Survey. Chin Med J (Engl). 2015;128(21):2843–2850. 26521779 10.4103/0366-6999.168017 PMC4756878

[5] Kubo T, Ueshima K, Saito M, et al. Clinical and basic research on steroid-induced osteonecrosis of the femoral head in Japan. J Orthop Sci. 2016;21(4):407–413. 27062553 10.1016/j.jos.2016.03.008

[6] Luo P, Gao F, Han J, et al. The role of autophagy in steroid necrosis of the femoral head: a comprehensive research review. Int Orthop. 2018;42(7):1747–1753. 29797168 10.1007/s00264-018-3994-8

[7] Murata M, Kumagai K, Miyata N, et al. Osteonecrosis in stroke-prone spontaneously hypertensive rats: effect of glucocorticoid. J Orthop Sci. 2007;12:289–295. 10.1007/s00776-007-1129-y 17530382

[8] Drescher W, Lohse J, Varoga D, et al. Enhanced constriction of supplying arteries–a mechanism of femoral head necrosis in Wistar rats? Ann Anat. 2010;192:58–61. 10.1016/j.aanat.2009.09.006 19880298

[9] Li Z, Zhao D, Wang B. ABCB1 gene polymorphisms and glucocorticoid-induced avascular necrosis of the femoral head susceptibility: a meta-analysis. Med Sci Monit. 2014;20:2811–2816. 25544111 10.12659/MSM.891286 PMC4285923

[10] Zhang Q, Jin L LVJ. Role of coagulopathy in glucocorticoid-induced osteonecrosis of the femoral head. J Int Med Res. 2018;46:2141–2148. 10.1177/0300060517700299 PMC6023042 28459353

[11] Qu Y, Liu Y, Li R. FSTL1 Promotes Inflammatory Reaction and Cartilage Catabolism through Interplay with NFκB Signaling Pathways in an In Vitro ONFH Model. Inflammation. 2019;42(4):1491–1503. 31011927 10.1007/s10753-019-01012-2

[12] Chen B, Liu Y, Cheng L. IL-21 Enhances the Degradation of Cartilage Through the JAK-STAT Signaling Pathway During Osteonecrosis of Femoral Head Cartilage. Inflammation. 2018;41(2):595–605. 29247327 10.1007/s10753-017-0715-1

[13] Adapala NS, Yamaguchi R, Phipps M, et al. Necrotic Bone Stimulates Proinflammatory Responses in Macrophages through the Activation of Toll-Like Receptor 4. Am J Pathol. 2016;186(11):2987–2999. 27648614 10.1016/j.ajpath.2016.06.024

[14] Rabquer BJ, Tan GJ, Shaheen PJ, et al. Synovial inflammation in patients with osteonecrosis of the femoral head. Clin Transl Sci. 2009;2(4):273–278. 20443906 10.1111/j.1752-8062.2009.00133.x PMC2925228

[15] Cai W, Li H, Zhang Y, et al. Identification of key biomarkers and immune infiltration in the synovial tissue of osteoarthritis by bioinformatics analysis. PeerJ. 2020;8:e8390. 31988808 10.7717/peerj.8390 PMC6970550

[16] Yao Y, Xie S, Wang F. Identification of key genes and pathways in chronic rhinosinusitis with nasal polyps using bioinformatics analysis. Am J Otolaryngol. 2019;40:191–196. 30661889 10.1016/j.amjoto.2018.12.002

[17] Xue G, Hua L, Zhou N, et al. Characteristics of immune cell infiltration and associated diagnostic biomarkers in ulcerative colitis: results from bioinformatics analysis. Bioengineered. 2021;12(1):252–265. 33323040 10.1080/21655979.2020.1863016 PMC8291880

[18] Zhou G, Soufan O, Ewald J, et al. 3.0: a visual analytics platform for comprehensive gene expression profiling and meta-analysis. Nucleic Acids Res. 2019;47(W1):W234–w41. 30931480 10.1093/nar/gkz240 PMC6602507

[19] Zhou Y, Zhou B, Pache L, et al. Metascape provides a biologist-oriented resource for the analysis of systems-level datasets. Nat Commun. 2019;10(1):1523. 30944313 10.1038/s41467-019-09234-6 PMC6447622

[20] Miao YR, Zhang Q, Lei Q, et al. ImmuCellAI: a Unique Method for Comprehensive T-Cell Subsets Abundance Prediction and its Application in Cancer Immunotherapy. Adv Sci (Weinheim, Baden-Wurttemberg, Germany). 2020;7:1902880. 10.1002/advs.201902880 PMC7141005 32274301

[21] Chin CH, Chen SH, Wu HH, et al. cytoHubba: identifying hub objects and sub-networks from complex interactome. BMC Syst Biol. 2014;8(Suppl 4):S11. 25521941 10.1186/1752-0509-8-S4-S11 PMC4290687

[22] Subramanian A, Narayan R, Corsello SM, et al. A Next Generation Connectivity Map: L1000 Platform and the First 1,000,000 Profiles. Cell. 2017;171(6):1437–52.e17. 29195078 10.1016/j.cell.2017.10.049 PMC5990023

[23] Irisa T, Yamamoto T, Miyanishi K, et al. Osteonecrosis induced by a single administration of low-dose lipopolysaccharide in rabbits. Bone.** **2001;28:641–649. 10.1016/s8756-3282(01)00460-4 11425653

[24] Feldman AT, Wolfe D. Tissue processing and hematoxylin and eosin staining. Methods Mol Biol. 2014;1180:31–43. 25015141 10.1007/978-1-4939-1050-2_3

[25] Chen H, Fang C, Zhi X, et al. Neobavaisoflavone inhibits osteoclastogenesis through blocking RANKL signalling-mediated TRAF6 and c-Src recruitment and NF-κB, MAPK and Akt pathways. J Cell Mol Med. 2020;24(16):9067–9084. 32604472 10.1111/jcmm.15543 PMC7417698

[26] Sugano N, Atsumi T, Ohzono K, et al. The 2001 revised criteria for diagnosis, classification, and staging of idiopathic osteonecrosis of the femoral head. J Orthop Sci. 2002;7:601–605. 10.1007/s007760200108 12355139

[27] Wang XY, Hua BX, Jiang C, et al. Serum Biomarkers Related to Glucocorticoid-Induced Osteonecrosis of the Femoral Head: a Prospective Nested Case-Control Study. J Orthop Res. 2019;37(11):2348–2357. 31254413 10.1002/jor.24400

[28] Cosman F, Eriksen EF, Recknor C, et al. Effects of intravenous zoledronic acid plus subcutaneous teriparatide [rhPTH(1-34)] in postmenopausal osteoporosis. J Bone Miner Res. 2011;26(3):503–511. 20814967 10.1002/jbmr.238

[29] Yuan L, Li W, Tian ZB, et al. Predictive role of cytokines IL-10, IL-12 and TNF-α gene polymorphisms for the development of osteonecrosis of the femoral head in the Chinese Han population. Cell Mol Biol (Noisy-le-grand). 2017;63(9):144–149. 28980933 10.14715/cmb/2017.63.9.23

[30] Miao Q, Hao S, Li H, et al. Expression of osteoprotegerin, RNAK and RANKL genes in femoral head avascular necrosis and related signaling pathway. Int J Clin Exp Pathol. 2015;8:10460–10467. 26617755 PMC4637570

[31] Okazaki S, Nishitani Y, Nagoya S, et al. Femoral head osteonecrosis can be caused by disruption of the systemic immune response via the toll-like receptor 4 signalling pathway. Rheumatology (Oxford). 2009;48(3):227–232. 10.1093/rheumatology/ken462 19129349

[32] Tian L, Wen Q, Dang X, et al. Immune response associated with Toll-like receptor 4 signaling pathway leads to steroid-induced femoral head osteonecrosis. BMC Musculoskelet Disord. 2014;15(1):18. 24428851 10.1186/1471-2474-15-18 PMC3904683

[33] Fang S, Li Y, Chen P. Osteogenic effect of bone marrow mesenchymal stem cell-derived exosomes on steroid-induced osteonecrosis of the femoral head. Drug Des Devel Ther. 2019;13:45–55. 10.2147/DDDT.S178698 PMC6305133 30587927

[34] Kuang MJ, Xing F, Wang D, et al. CircUSP45 inhibited osteogenesis in glucocorticoid-induced osteonecrosis of femoral head by sponging miR-127-5p through PTEN/AKT signal pathway: experimental studies. Biochem Biophys Res Commun. 2019;509(1):255–261. 30579595 10.1016/j.bbrc.2018.12.116

[35] Zha X, Sun B, Zhang R, et al. Regulatory effect of microRNA-34a on osteogenesis and angiogenesis in glucocorticoid-induced osteonecrosis of the femoral head. J Orthop Res. 2018;36:417–424. 28543623 10.1002/jor.23613

[36] Longobardi L, Temple JD, Tagliafierro L, et al. Role of the C-C chemokine receptor-2 in a murine model of injury-induced osteoarthritis. Osteoarthritis Cartilage. 2017;25(6):914–925. 27856294 10.1016/j.joca.2016.11.004 PMC5430000

[37] Raghu H, Lepus CM, Wang Q, et al. CCL2/CCR2, but not CCL5/CCR5, mediates monocyte recruitment, inflammation and cartilage destruction in osteoarthritis. Ann Rheum Dis. 2017;76(5):914–922. 27965260 10.1136/annrheumdis-2016-210426 PMC5834918

[38] Wojdasiewicz P, Turczyn P, Dobies-Krzesniak B, et al. Role of CX3CL1/CX3CR1 Signaling Axis Activity in Osteoporosis. Mediators Inflamm. 2019;2019:7570452. 31780870 10.1155/2019/7570452 PMC6875359

[39] Onuora S. Osteoarthritis: a role for CXCR2 signalling in cartilage homeostasis. Nat Rev Rheumatol. 2014;10(10):576. 10.1038/nrrheum.2014.148 25179389

[40] Sherwood J, Bertrand J, Nalesso G, et al. A homeostatic function of CXCR2 signalling in articular cartilage. Ann Rheum Dis. 2015;74(12):2207–2215. 25135253 10.1136/annrheumdis-2014-205546 PMC4680121

[41] Liu W, Wang P, Xie Z, et al. Abnormal inhibition of osteoclastogenesis by mesenchymal stem cells through the miR-4284/CXCL5 axis in ankylosing spondylitis. Cell Death Dis. 2019;10(3):188. 30804325 10.1038/s41419-019-1448-x PMC6389901

[42] Kamal D, Trăistaru R, Kamal CK, et al. Macrophage response in patients diagnosed with aseptic necrosis of the femoral head presenting different risk factors. Rom J Morphol Embryol. 2015;56:163–168. 25826501

[43] Puissant E, Boonen M. Monocytes/Macrophages Upregulate the Hyaluronidase HYAL1 and Adapt Its Subcellular Trafficking to Promote Extracellular Residency upon Differentiation into Osteoclasts. PloS One. 2016;11(10):e0165004. 27755597 10.1371/journal.pone.0165004 PMC5068775

[44] Kasonga A, Kruger MC, Coetzee M. Activation of PPARs Modulates Signalling Pathways and Expression of Regulatory Genes in Osteoclasts Derived from Human CD14+ Monocytes. Int J Mol Sci. 2019;20(7):1798. 10.3390/ijms20071798 PMC6479901 30979019

[45] Wang C, Wang X, Xu XL, et al. Bone microstructure and regional distribution of osteoblast and osteoclast activity in the osteonecrotic femoral head. PloS One. 2014;9(5):e96361. 24800992 10.1371/journal.pone.0096361 PMC4011745

[46] Nishii T, Sugano N, Miki H, et al. Does alendronate prevent collapse in osteonecrosis of the femoral head? Clin Orthop Relat Res. 2006;443:273–279. 16462451 10.1097/01.blo.0000194078.32776.31

[47] Lin S, Lee WYW, Huang M, et al. Aspirin prevents bone loss with little mechanical improvement in high-fat-fed ovariectomized rats. Eur J Pharmacol. 2016;791:331–338. 27615444 10.1016/j.ejphar.2016.09.018

[48] Tao ZS, Wu XJ, Zhou WS, et al. Local administration of aspirin with β-tricalcium phosphate/poly-lactic-co-glycolic acid (β-TCP/PLGA) could enhance osteoporotic bone regeneration. J Bone Miner Metab. 2019;37(6):1026–1035. 31076895 10.1007/s00774-019-01008-w

[49] Albers A, Carli A, Routy B, et al. Treatment with acetylsalicylic acid prevents short to mid-term radiographic progression of nontraumatic osteonecrosis of the femoral head: a pilot study. Can J Surg J Canadien De Chirurgie. 2015;58:198–205. 10.1503/cjs.016814 PMC4447515 26011853

