# Supplementary material for: Comprehensive analysis of pivotal biomarkers, immune cell infiltration and therapeutic drugs for steroid-induced osteonecrosis of the femoral head
Source: Bioengineered. 2021 Sep 7;12(1):5971–84. doi: 10.1080/21655979.2021.1972081 (PMC8815624; doi:10.1080/21655979.2021.1972081)
Supplement: Supplemental Material [file KBIE_A_1972081_SM3078.zip › supplementary/Supplementary Table S1.docx]

| **Table 1.** The primer sequences used for quantitative real-time PCR. | | |
| --- | --- | --- |
| Gene | Forward primer sequence  (5’→3’) | Reverse primer sequence  (5’→3’) |
| CCR1 | GAAATCCAAAGCTGTCCGT | CCAAATGTCTGCTCTGCTC |
| CCR2 | CTCCGCCTTCACTTTCTG | TGTTCAGCTTGTGGCTTG |
| CCR3 | GGGGCCTCAGTATTCGAT | AGGGACAAGGGTGAAGATG |
| CXCR1 | CCTGCCCTTCTTCCTTTT | ACACCATCCGCCATTTT |
| CXCR2 | CCTGTCTTACTTTTCCGAAGGAC | TTGCTGTATTGTTGCCCATGT |
| FPR2 | TGGACATCAACCTCTTTGG | TCCGACGATCACCTTCA |
| CXCL5 | CTGCGTTGCGTTTGTTTAC | ATTTCCTTCCCGTTCTTCA |
| PF4 | CTGAAGAAGATGGGGACCT | GGCTATGAGTTGGGCAGT |
| P2RY13 | CTCCTCCACCTTCATCATCT | AACGACACACAAAAGCTCTG |
| HCAR2 | AGGTATTTCCGGGTGGTC | TGGATCGGCATCTTCTTC |
| RUNX2 | GCCGGGAATGATGAGAAC | TGGGGAGGATTTGTGAAGA |
| RANKL | GAAAGGAGGGAGCACGA | GGGTTGGACACCTGAATG |
| PTGS2 | AAATGCTGGTGTGGAAGGT | TTGTTGCTCTAGGCTTTGCT |
| GAPDH | ACAACTTTGGTATCGTGGAAGG | GCCATCACGCCACAGTTTC |
