# Supplementary material for: Comprehensive analysis of pivotal biomarkers, immune cell infiltration and therapeutic drugs for steroid-induced osteonecrosis of the femoral head
Source: Bioengineered. 2021 Sep 7;12(1):5971–84. doi: 10.1080/21655979.2021.1972081 (PMC8815624; doi:10.1080/21655979.2021.1972081)
Supplement: Supplemental Material [file KBIE_A_1972081_SM3078.zip › supplementary/Supplementary Table S2.docx]

Gene

CHI3L1

KRT23

LGALS2

CCR3

CFD

OLIG1

CXCR2

SDPR

CPVL

CD163

HAL

PELI1

ADGRE3

CXCR2

LYZ

IFIT2

VCAN

DPEP2

MPEG1

TGFBI

CPPED1

RGS2

FCER1A

DUSP6

SLC45A4

CST3

CXCR1

SIGLEC7

HSPA6

IFIT1

FFAR2

TREM1

SGK1

FAM198B

WLS

CD14

HCAR2

PI3

CCR2

PECAM1

PTGS2

ST6GALNAC2

CD302

PTAFR

AMICA1

CLEC12A

MX1

TMCC3

OGFRL1

NRBF2

SLC7A7

MYO1F

VMP1

P2RY13

IGKC

HSPA6

CTSS

FRAT2

RNASE6

ST3GAL6

TNFSF13B

IGKV1-39

IL13RA1

IGHG1

HERC5

LILRB2

SORL1

FAM174A

NCF2

FRY

TLR2

NRGN

TLR8

HVCN1

SULF2

TNFRSF10C

HLA-DRB1

PLEKHO1

HCK

TYROBP

CLEC4A

CEBPD

FGL2

LOC254896

FCN1

F2RL1

IGF2R

AOAH

MX2

RGS18

SNX10

MPPE1

SUSD6

ATP6V1B2

C10orf54

TUBB1

S1PR3

LYN

TNFSF10

LRRK2

CEP19

ABCG1

MNDA

EPB41L3

IGLL5

LILRB1

MOSPD2

LST1

MS4A7

CLC

SHTN1

RNASET2

LPAR2

IGK

LAMP2

IFIT3

NUMB

PF4

RTN1

TMEM71

SMPDL3A

CCR1

ARHGAP26

FPR2

CD46

MXD1

TMEM170B

NLRP1

CBWD1

IFI30

IGHG1

ARRDC3

HLA-DRB1

LOC728392

LRRN3

PLEKHO2

NADK

AKIP1

SLC15A4

KCTD12

TLR1

XPO6

NAGK

MIR21

SERPINA1

LILRB1

RPS6KA5

TLE3

LOC105369243

PSAP

NCF1

LOC101928143

MSRB1

MOB3A

TIGD3

IL17RA

PAK1

MTMR11

CEBPA

TSEN34

FCGR3A

ABHD3

HLA-DRB1

CNTNAP3

ATP2B1

PLAGL1

TMEM154

CMTM2

NCF4

CD36

IMPA2

TLR7

APLP2

LOC100996763

SMCHD1

STEAP4

RHOQ

ALDH2

ARAP1

GNG11

IFNGR2

ALDH1A1

APOBEC3A

IKBIP

MS4A6A

CARD16

UBE2D1

IFNGR1

LCP2

BASP1

TXNIP

FAM65B

F13A1

PKN2

LOC100131541

FCGR2A

RNF130

TYK2

KCNJ15

EVI2B

PIGX

CD300A

CXCL5

S100A11

AQP9

CARD16

FAM49A

IRF8

PROK2

PLXNC1

LILRA6

CD93

CSF3R

ND1

IGLL1

KCNH2

CETN2

TK1

S100A16

UBE2F

RGCC

HBQ1

DPM2

PXDN

MCOLN1

TCF3

FBXL4

CDKN2C

NEURL1B

PSMF1

FAM83A

ZER1

RUNDC3A

LTF

E2F2

AP2B1

FHL2

MAP4K5

PBX1

HK1

TRIM10

DNAJA4

CDC34

TNS1

FIS1

FKBP8

STK11

TMEM200B

HP

EIF2AK1

HBM

RNF10

LRRC75A

IGF2BP2

ACP5

MSI2

IFRD2

GABARAPL2

FOXO3

RHCE

HDGF

ELOF1

TERF2IP

AGO2

TSPO2

KEL

MBNL3

KAT2B

GID4

CD177

ANKRD9

RNF123

PLEK2

SEC62

SESN3

GSPT1

CMAS

YIPF6

STRADB

FKBP1B

NTAN1

ELANE

DCAF12

NME4

GCLC

SMIM5

VTI1B

JAZF1

BCL2L1

MOSPD1

MYL6B

SNX3

FOXO3

OLFM4

NFIX

ST6GALNAC4

PCTP

GYPE

EPB41

CDKL1

POLR1D

OSBP2

DMTN

PAQR9

SLC7A5

PGF

BSG

FAM104A

ABCG2

C9orf78

MPP1

CHPT1

SLC6A8

SMOX

TBCEL

OR2W3

POC1B

STOM

C7orf73

TMEM56

HRSP12

TMEM86B

FAM210B

RAB2B

TFR2

RAD23A

CNN3

CLIC2

HIST1H1C

CREG1

ELOVL6

SMIM24

BNIP3L

ALAS2

GPR146

ZNF23

HES6

TSPAN5

RNF14

SRRD

PNP

C20orf194

DCUN1D1

RPIA

TPGS2

ARG1

RBM38

IGF2

MMP8

TFDP1

ARHGEF12

KANK2

C9orf40

KLC3

SLC1A5

SLC2A1

DNAJC6

ARG2

NSUN3

DNTT

REXO2

TMEM158

ABCC13

HMBS

ACSM3

YPEL4

GMPR

TRAK2

EPB42

DYRK3

RHD

TFDP2

SLC6A10P

ACSL6

TCP11L2

CTSG

USP12

UBE2H

TMOD1

RNF11

SPTA1

GLRX5

ANK1

MXI1

PRDX2

TRIM58

PITHD1

ALDH5A1

TSTA3

GYPB

GADD45A

SELENBP1

SNCA

RHAG

ARL4A

SLC4A1

THEM5

CA2

TMCC2

RIOK3

SIAH2

KLF1

PIP5K1B

BBOF1

ISCA1

NUDT4

TSPAN7

FHDC1

CISD2

FECH

AHSP

HBD

YOD1

CTNNAL1

CA1

RNF182

IFI27

FAM46C

KRT1

SLC14A1

RHCE

XK

HEPACAM2

GYPA

HEMGN

IFIT1B

RAP1GAP

BPGM

EIF1AY
